# Smoking is associated with increased risk of myeloproliferative neoplasms: A general population‐based cohort study

**DOI:** 10.1002/cam4.1815

**Published:** 2018-10-14

**Authors:** Kasper M. Pedersen, Marie Bak, Anders L. Sørensen, Ann‐Dorthe Zwisler, Christina Ellervik, Morten K. Larsen, Hans C. Hasselbalch, Janne S. Tolstrup

**Affiliations:** ^1^ Department of Hematology Zealand University Hospital, University of Copenhagen Roskilde Denmark; ^2^ Institute for Inflammation Research, Centre for Rheumatology and Spine Diseases, Rigshospitalet Copenhagen University Hospital Copenhagen Denmark; ^3^ REHPA, Danish Knowledge Centre for Rehabilitation and Palliative Care University of Southern Denmark and Odense University Hospital Nyborg Denmark; ^4^ Division of Clinical Medicine, Faculty of Health and Medical Sciences University of Copenhagen Copenhagen Denmark; ^5^ Department of Laboratory Medicine Boston Children’s Hospital & Harvard Medical School Boston Massachusetts, USA; ^6^ Department of Science and Environment University of Roskilde Roskilde Denmark; ^7^ National Institute of Public Health, University of Southern Denmark Copenhagen Denmark

**Keywords:** epidemiology, essential thrombocythemia, myeloproliferative neoplasms, polycythemia vera, primary myelofibrosis, tobacco smoking

## Abstract

**Background:**

Former studies on smoking as a risk factor for Philadelphia‐negative myeloproliferative neoplasms (MPNs) have mainly been carried out in women's cohorts and studies with various definitions of MPNs. Herein, we conducted a cohort study with register‐based follow‐up of a general population from Denmark, to validate and substantiate prior observations.

**Methods:**

In the Danish Health Examination Survey cohort, we used the Cox proportional‐hazards model adjusted for age, sex, body mass index, and level of education, to calculate hazard ratios (HRs), to investigate, whether daily smokers or occasional/ex‐smokers had an increased risk of MPNs compared to never‐smokers.

**Results:**

From the time of data collection (September 2007 to October 2008) until 1 January 2015, 70 individuals were diagnosed with MPNs among 75 896 study participants. Similar results were observed in both the age and sex adjusted analysis and the multivariable analysis. The multivariable HR of any MPN diagnosis for daily smokers was 2.5 (95% CI: 1.3‐5.0). For essential thrombocytosis, polycythemia vera, myelofibrosis, and MPN‐unclassified, the HRs were 1.8 (95% CI: 0.5‐5.8), 1.7 (95% CI: 0.5‐5.8), 4.3 (95% CI: 0.9‐19), and 6.2 (95% CI: 1.5‐25), respectively. Among occasional/ex‐smokers the corresponding HRs were 1.9 (95% CI: 1.1‐3.3), 1.5 (95% CI: 0.6‐3.7), 0.8 (95% CI: 0.3‐2.4), 0.9 (95% CI: 0.2‐4.4), and 6.2 (95% CI: 1.8‐21). Participants, who smoked >15 g/day, had an overall HR of 3.4 (95% CI: 1.4‐8.2) for any MPN diagnosis, while participants who smoked ≤15 g/day, had an overall HR of 2.1 (95% CI: 0.9‐4.7).

**Conclusion:**

Smoking was associated with MPN development when comparing smokers and never‐smokers. Further studies investigating smoking in MPNs are warranted to substantiate our findings.

## 1 INTRODUCTION

Smoking is associated with a chronic low‐grade inflammatory state as evidenced by increased levels of several pro‐inflammatory cytokines, in vivo activation of leukocytes and platelets, endothelial dysfunction, and systemic oxidative stress.^1^ Additionally, smoking is associated with a pattern of persistent chronic hypoxic hyperstimulation of myeloid cells assessed by elevated levels of the hematocrit, leukocytosis, monocytosis, and occasionally thrombocytosis.^1^ Interestingly, these inflammatory components and blood cell indices are also a typical presentation of the Philadelphia‐negative chronic myeloproliferative neoplasms (MPNs), encompassing essential thrombocythemia (ET), polycythemia vera (PV), myelofibrosis (MF), and unclassifiable MPNs (MPN‐U).^2^

The biological continuum of MPNs—from ET through PV to the advanced myelofibrosis state—has been proposed to constitute “A Human Inflammation Model for Cancer Development.”^3^ According to this model, chronic inflammation, which is also generated by the neoplastic cells themselves, may be an important driver of clonal evolution, premature atherosclerosis, and development of second cancers.^4^, ^5^ The perspectives of smoking as a contributing factor for MPNs have most recently been thoroughly described by Hasselbalch,^3^ including previous important epidemiological studies demonstrating an association of smoking and MPNs.^6^, ^7^, ^8^ Consequently, it has been highlighted that smokers and patients with MPNs share features of an increased inflammatory burden, including, upregulation of important molecular pathways and transcriptions factors^3^, ^9^ These shared factors, all of which are vigorously amplified,^10^, ^11^, ^12^, ^13^ are known to be involved in tumorigenesis and cancer progression.^14^ Owing to these similarities and the model of chronic inflammation and its role in the clonal evolution and progression of MPNs, it is tempting to suggest smoking as one of several potent inflammatory stimuli potentially triggering and driving the MPN‐clone.

This cohort study with register‐based follow‐up of selected individuals from the general population in Denmark, aimed to investigate whether smokers had an increased risk of MPNs compared to never‐smokers.

## 2 METHODS

We used data from the population‐based study “The Danish Health Examination Survey” (DANHES).^15^ DANHES conformed to the principles of the Declaration of Helsinki and written informed consent was obtained from all participants. The National Institute of Public Health, University of Southern Denmark, and the Danish Data Protection Agency gave their permission to carry out this specific study (J.nr 2007‐54‐0017). An approval from the Danish Health Authority and the National Committee on Health Research Ethics was not needed, as registry‐based studies are exempted.

In DANHES, all citizens between 18 and 99 years of age from 13 of the 98 municipalities in Denmark (n = 538 497) were invited by letter to fill out two internet‐based questionnaires; a basic questionnaire containing questions on sociodemographic, health behavior including smoking status, self‐reported health, and living conditions plus a supplementary questionnaire concerning diet. A total of 76 484 citizens, corresponding to 14% of the general population in the respective municipalities, filled out the questionnaires. Data were collected between September 2007 and October 2008.

Smoking status was reported as never, daily, current but not daily (reported in categories of at least once per week but not daily, once per month, or rarer than once per month), and former smoking. Participants were categorized as never‐smokers, occasional smokers (ie current non‐daily) combining ex‐smokers, and daily smokers. Occasional and ex‐smokers were combined due to a relatively low number of cases in the two categories. Amount of smoking was reported separately for cigarettes, cheroots and cigars (in numbers per day), and pipe tobacco (in grams per week). Assuming 1 cigarette to be equivalent to 1 g of tobacco, 1 cheroot to 3 g of tobacco, 1 cigar to 5 g of tobacco, and dividing the reported grams of weekly pipe tobacco with 7, a measure of daily amount of smoking in g/day was calculated as a sum of the tobacco from the individual types. Cumulative smoking was calculated in pack‐years based on information on duration of smoking and amount of consumed tobacco: one pack‐year was 20 g smoked daily for one year. Participants with no available data on smoking status were excluded from the analyses. Level of education as well as body mass index (BMI) was categorized into three groups (<10, 10‐12, and ≥12 years and <25, 25‐30, and >30 kg per square meter, respectively).

Participants’ unique civil registration number enabled individual level linkage of data with national registries.^16^ The civil registration numbers given at birth or immigration to all Danish citizens ensured that different longitudinal measures for all participants could be obtained, including information on all in and out hospital visits and associated diagnosis codes from the Danish National Patient Registry,^17^ including information on MPN diagnosis, as well as information on vital status and emigration recorded in the Danish Civil Registration System.^18^ We used the following codes from the International Classification of Diseases to identify patients with MPNs; ET: 8th revision = 287.29, 10th revision = D47.3, D75.2, PV: 8th revision = 208.99, 10th revision = D45, MF: 8th revision = 209, 10th revision = D47.4, C94.4, C94.5, and MPN‐U: 10th revision = D47.1. Participants, who had any MPN diagnosis prior to baseline, were excluded. Any records of either ET, PV, MF, or MPN‐U from baseline until end of follow‐up were defined as MPN events in this study. We followed everyone from baseline to either time of MPN diagnosis, loss to follow‐up, death, or end of follow‐up at 1 January 2015. To increase the accuracy of newly recorded MPNs, participants who were subsequently diagnosed with “cytosis by other causes” (secondary polycythemia; 10th revision: D75.1) were excluded to avoid possible misclassification bias; a method previously described.^19^

### 2.1 Statistical analysis

We calculated hazard ratios (HR) and 95% confidence intervals (95% CI) using Cox proportional‐hazards models with delayed entry. Age (in days) was used as the underlying time axis to ensure maximal adjustment for confounding by age.^20^ We examined the Cox proportional‐hazards assumption by plots of log(−time) against log(−log(survival probability)) and by introducing interaction terms between age and alcohol intake in the model, with no violations detected. We performed both an age and sex adjusted analysis comparing daily smokers and occasional/ex‐smokers with never‐smokers and a multivariable analysis adjusted for age, sex, and level of education as well as BMI, as studies have shown that obesity is independently associated with inflammation and MPNs.^21^, ^22^, ^23^ Information on height or weight (and thus BMI) was missing for four percent of the participants. To avoid reducing the sample size, a dummy variable indicating missing BMI value was constructed and used in analyses. Former studies have not been able to show an association with alcohol, why we refrained from adjusting for this.^6^, ^7^ Daily smokers were further divided in two groups based on daily amount of smoking (>15 g/day and ≤15 g/day) and compared with never‐smokers, to look at the effect of amount of daily smoking on the risk estimates. Similarly, we examined the risk estimate per 10 pack‐years of smoking to investigate the effect of cumulative smoking in ever‐smokers. Descriptive statistics were presented as medians for continuous variables and as frequencies and percentages for categorical variables. Results were considered statistically significant at the *P* < 0.05 level. All analyses were performed using SAS 9.4 (The SAS Institute, Cary, NC, USA).

## 3 RESULTS

In total, 76 484 survey participants were potentially eligible for inclusion. Of those, 588 participants were subsequently excluded; 510 participants due to missing information on smoking status, 54 participants with a MPN diagnosis prior to data collection, 23 participants due to errors in the registration key, and one participant due to a diagnosis of “secondary cytosis”. Thus, a total of 75 896 persons were included in the final analyses, including 45 030 (59.7%) women and 30 866 (40.7%) men. Median age was 49.0 years (range 19‐99 years) for women and 52.8 years (range 18‐99) for men. During the follow‐up period, 2443 participants died and 580 were lost to follow‐up; 576 emigrated and 4 had their civil registration numbers changed. Among all women and men, 13.3% and 14.6% were daily smokers, stratification on daily amount of tobacco consumption can be seen in Table 1, and 34.4% and 40.5% were occasional/ex‐smokers, respectively. The total follow‐up time was 518 977 person‐years, and the mean time of follow up was 6.8 years (SD = 0.9 years).

**Table 1:** Baseline characteristics of the population from the Danish Health Examination Survey study (2007‐2008)

Characteristics	Women	Men
Participants, n	45 030	30 866
Age, years, median (range)	49.0 (19‐99)	52.8 (18‐99)
Smoking status, n (%)		
Never	23 561 (52.3)	13 850 (44.9)
Occasional/Ex	15 492 (34.4)	12 509 (40.5)
Daily	5977 (13.3)	4507 (14.6)
≤15 g/day	4187 (9.3)	2580 (8.4)
>15 g/day	1790 (4.0)	1927 (6.2)
Pack‐years, median (10th, 90th percentile)		
Occasional/Ex	7.8 (0, 30)	14.0 (0, 44)
Daily	16.0 (3.8, 39)	21.6 (4.8, 50.7)
Marital status, married, n (%)	26 430 (58.7)	19 969 (64.7)
Educational level, n (%)		
<10 yr	6706 (14.9)	4790 (15.5)
10‐12 yr	8404 (18.7)	7023 (22.8)
≥12 yr	29 920 (66.4)	19 053 (61.7)
Self‐rated health^a^, low, n (%)	11 087 (24.6)	7487 (24.3)
Body mass index, n (%)^b^		
<25 kg/m^2^	27 469 (61.0)	13 656 (44.2)
25‐30 kg/m^2^	11 135 (24.7)	12 629 (40.9)
>30 kg/m^2^	4614 (10.3)	3259 (10.6)

a Self‐rated health was assessed by the question “in general, would you say that your health is excellent, very good, good, fair, or poor?,” and low self‐rated health was defined by combining the latter three categories.

b Numbers do not sum up to 100% due to missing information.

In total, 70 new cases of MPNs were diagnosed among the survey participants during the period of follow‐up; 41 were women and 29 were men. The distribution of MPNs was 23 ET, 17 PV, 10 MF, and 27 MPN‐U. Incidence rates are available in Table 2. Median age at MPN diagnosis was 63.0 years (range 21‐86 years). Similar results, shown in Table 1, were observed in both the age and sex adjusted analysis and the multivariable analysis. The multivariable HR of any MPN during the study period for daily smokers was 2.5 (95% CI: 1.3‐5.0) and 1.9 (95% CI: 1.1‐3.3) for occasional/ex‐smokers. Stratified by MPN subtype, only the risk estimate of MPN‐U was significantly increased with a HR of 6.2 (95% CI: 1.5‐25) among daily smokers and 6.2 (95% CI: 1.8‐21) among occasional/ex‐smokers. The HRs of all MPN subtypes are presented in Table 2. For daily amount of smoking, analysis showed that participants who smoked >15 g/day had a HR of 3.4 (95% CI: 1.4‐8.2), while participants who smoked ≤15 g/day had a HR of 2.1 (95% CI: 0.9‐4.7) for any MPN compared to never‐smokers. For cumulative smoking, analysis showed a HR of 1.14 (95% CI: 1.06‐1.22, *P*‐value for trend = 0.0005) per 10 pack‐years of smoking in ever‐smokers. The HR remained significant after adjusting for smoking status, HR = 1.10 (95% CI: 1.00‐1.21, *P*‐value for trend = 0.05).

**Table 2:** Hazard ratios of myeloproliferative neoplasms by smoking status with never‐smokers as the reference group. Age and sex‐adjusted and multivariable analysis

	MPN, total	ET	PV	MF	MPN‐U
Person‐years at risk, yr	518 977	519 110	519 144	519 084	519 118
Events, n					
Never‐smokers	20	9	7	3	3
Occasional/ex‐smokers	35	10	6	3	18
Daily smokers	15	4	4	4	6
Total	70	23	17	10	27
Incidence rate per 100 000 person‐years	13.5	4.4	3.3	1.9	5.2
Smoking status, HR (95% CI)					
Age and sex‐adjusted					
Occasional/Ex	1.9 (1.1‐3.3)	1.5 (0.6‐3.8)	0.8 (0.3‐2.4)	0.9 (0.2‐4.4)	6.0 (1.8‐21)
Daily	2.6 (1.3‐5.1)	1.7 (0.5‐5.6)	1.7 (0.5‐6.0)	4.4 (1.0‐20.0)	6.9 (1.7‐28)
Multi‐adjusted ῲ					
Occasional/Ex	1.9 (1.1‐3.3)	1.5 (0.6‐3.7)	0.8 (0.3‐2.4)	0.9 (0.2‐4.4)	6.2 (1.8‐21)
Daily	2.5 (1.3‐5.0)	1.8 (0.5‐5.8)	1.7 (0.5‐5.8)	4.3 (0.9‐19)	6.2 (1.5‐25)

ET, essential thrombocytosis; MF, myelofibrosis; MPN, myeloproliferative neoplasms; MPN‐U, unclassifiable myeloproliferative neoplasm; PV, polycythemia vera; ῲ, Adjusted for age, sex, body mass index, and education level.

## 4 DISCUSSION

In this study with register‐based follow‐up of the DANHES survey participants, we found smoking to be a significant risk factor for the development of MPNs. The risk of MPNs was 2.5‐fold higher among daily smokers and 1.9‐fold higher among occasional/ex‐smokers compared to never‐smokers during follow‐up in the multivariable analyses. For the subtype analysis, daily and occasional/ex‐smokers had 6.2‐fold higher risks of MPN‐U compared to never‐smokers. Concerning ET, PV, and MF, the HRs for daily smokers were all above one, although not statistically significant. However, caution should be taken when interpreting these results as they are likely affected by the low number of cases because of a relatively short follow‐up period combined with the fact that MPNs are rare diseases,^24^ with approximately 500 patients being diagnosed each year in Denmark, with a population of approximately 5.5 million people.^25^ The observed dose–response relationship with a HR of 1.10 for any MPN per 10 pack‐years of smoking, as well as the observed 3.4‐fold and 2.1‐fold (95% CI: 0.9‐4.7) increased risk of any MPN in participants who smoked >15 g/day and ≤15 g/day, respectively, support the proposed concept of smoking as a potent inflammatory stimulus driving the MPN development; however, the observed associations are not conclusive per se.

In line with the findings in this study, Kroll et al^6^ reported an increased risk of myeloproliferative diseases, including both classical MPNs, myelodysplastic syndrome, chronic myelogenous leukemia, and other myeloproliferative disorders, among smokers (relative risk of 1.42 (95% CI: 1.31‐1.55) per 10 cigarettes/day). However, this study did not investigate classical MPNs alone. Leal et al^7^ found that current smokers had an increased risk of all MPNs as well (relative risk of 1.72 [95% CI: 1.16‐2.56]). This showed mainly to be due to the risk of PV, as the risk of ET was nonsignificant. MF and MPN‐U were not investigated separately. In contrast to the present study, both studies were performed on middle‐aged women. Most recently, a Danish single‐institution case–control study found an association between history of smoking in a general MPN population compared to patients with chronic lymphocytic leukemia.^8^ It should be mentioned as well, that conflicting with the above‐mentioned results and the results in the present study, two earlier case–control studies could not find an association between smoking and risk of MPNs.^26^, ^27^ Pasqualetti et al^26^ pooled together CML (n = 69), idiopathic myelofibrosis (n = 11), PV (n = 8), and idiopathic thrombocythemia (n = 4), thereby increasing the heterogenicity and subsequently lowering the comparability with the present study. Mele et al^27^ only investigated cases of ET (n = 39) and smoking exposure was barely defined, thereby obscuring the transparency and conclusion of the study. Most recently, existing studies have been compiled in a meta‐analysis with 1 368 738 individuals and 2017 MPN cases.^28^ Pooled odds ratio for MPNs were 1.44 (95% CI: 1.33‐1.56) comparing smokers to nonsmokers; 1.10 (95% CI: 0.86‐1.41) comparing ex‐smokers to nonsmokers; and 1.30 (95% CI: 1.14‐1.49) comparing ever‐smokers to never smokers. In subgroup analysis, smokers had a statistically significantly increased risk of ET; however, odds ratios were above one for both PV and MF.

The association found is supported by the many molecular and cellular similarities between the consequences of smoking and MPNs and has most recently been described as “The Smoke‐MPN‐Cancer Loop.”^3^ One of many inflammatory cytokines increased in smokers and playing a role in MPN pathogenesis is tumor necrosis factor alpha, which, in addition to nuclear factor kappa beta (NF‐kβ) upregulation, has been shown to facilitate clonal expansion in JAK2‐V617F‐bearing cells.^7^, ^29^ NF‐kβ is also associated with increased production of transforming growth factor beta and vascular endothelial growth factor,^11^ both highly immunosuppressive cytokines that are elevated in the circulation in MPNs and markedly expressed in the bone marrow.^5^, ^30^ Both cytokines may induce qualitative and quantitative alterations in immune cells, thereby potentially impairing the functionality of these immune cells and consequently “tumor immune surveillance,” the ultimate outcome being expansion of the malignant clone.^4^, ^5^, ^30^ Smoking is also associated with increased hypoxia‐inducible factor signaling^12^ and interleukin 1‐beta secretion from mononuclear cells,^31^ leading to upregulation of nuclear factor erythoid‐2 expression in megakaryocytic cells and IL‐8 secretion.^10^, ^32^ These observations are indeed intriguing, as both are believed to play a vigorous role in MPN‐pathogenesis as well.^33^, ^34^

This cohort study, with focus on risk of MPNs in smokers, is, to our knowledge, the first in a general population including both women and men. Additionally, by including 75 896 individuals, it is the largest cohort study focusing isolated on smoking as a risk factor for classical MPNs as the comprehensive study by Kroll et al^6^ pooled myeloproliferative and myelodysplastic disorders among other diagnoses in their analyses. The main strengths in the study are the size of the cohort and the use of valid population‐based central administrative registries with minimal loss to follow‐up. We were able to include all MPNs among the DANHES participants as all MPN patients are followed at hospitals in Denmark and hospital diagnoses at Danish hospitals must be reported to the Danish National Patient Registry.^17^ In this context, diagnosis codes for hematological malignancies are found to be valid.^35^ Nevertheless, the study also has some limitations. Because MPNs can be difficult to diagnose and the MPN diagnoses in this study was not verified by information from medical files, we cannot exclude that a few participants could have been misclassified, for example, some heavy smokers could have been misclassified as having a MPN based upon a secondary cytosis or a reactive thrombocytosis. Although the impact of misclassified cases is hard to estimate without access to medical files, we are confident that it is minimal, because, in Denmark, a bone marrow biopsy is done routinely in all individuals suspected of having MPN. However, since patients who were diagnosed with MPNs and subsequently a “secondary cytosis” were excluded, we hereby tried to prevent this bias. Furthermore, we excluded 510 participants with missing information on smoking (<1%); in these participants, no MPN cases after follow‐up occurred. While selective missing bias cannot be ruled out, it does not seem that excluded participants had a higher risk as compared to participants with non‐missing information on smoking.

Former studies have also investigated risk factors for MPNs, and rather convincing associations have been found concerning familial MPNs and some autoimmune disorders.^36^ Also, associations with some occupations and exposures to different toxins are found, although conflicting evidence exists in this area.^36^ As we did not have access to this information and due to the possibility, that subtypes of MPNs may have various carcinogenesis and, thus, different risk factors, we cannot rule out that the results in the present study are affected by some degree of residual confounding. Furthermore, with surveys, selection and reporting bias is possible. DANHES reported that individuals who were unmarried, had the lowest level of education, or lowest category of income were under‐represented compared to the Danish population.^15^ Under‐reporting of smoking could have occurred in our cohort as 13.3% and 14.6% of women and men reported daily smoking. This contrasts with a survey made upon the request of the Danish Health Authority in 2008 on 4523 persons, in which they found 22.3% and 24.1% of the Danish women and men as daily smokers.^37^ It should also be mentioned that occasional and ex‐smokers were combined in the present study. The risk estimate for this combined group may be higher than the risk estimate would be for a group of pure ex‐smokers because of the contribution from occasional smokers. Lastly, as smoking exposure was only reported at the time of inclusion in DANHES, we were not able to account for smoking status at the time the MPN diagnosis was reported to the Danish National Patient Registry.

In conclusion, we have shown an increased risk of MPNs in smokers compared to never‐smokers in a cohort of selected individuals from the general population in Denmark. The results of this study support the growing evidence suggesting smoking to be a risk factor in the development of MPNs.

## CONFLICT OF INTEREST

None declared.
